# Prenatal diagnosis of congenital talipes equinovarus: favorable outcome is highly dependent on the absence of associated anomalies

**DOI:** 10.1590/1806-9282.20252156

**Published:** 2026-06-29

**Authors:** Gökhan Ünver, Sercan Serin, Ümmet Abur, Hüseyin Ekici, Handan Çelik, Miğraci Tosun

**Affiliations:** 1 Ondokuz Mayis University, Faculty of Medicine, Department of Obstetrics and Gynecology, Perinatology Unit – Samsun, Türkiye.; 2 Ondokuz Mayis University, Faculty of Medicine, Department of Medical Genetics – Samsun, Türkiye.

**Keywords:** Clubfoot, Congenital talipes equinovarus, Prenatal diagnosis, Ultrasonography

## Abstract

**OBJECTIVE:**

The aim of this study was to evaluate the demographic characteristics, prenatal findings, and outcomes of prenatally diagnosed congenital talipes equinovarus at a tertiary center.

**METHODS:**

A retrospective cohort study was conducted on 78 fetuses diagnosed with congenital talipes equinovarus between 2013 and 2023. Data on gestational age at diagnosis, laterality, associated anomalies, genetic results, and pregnancy outcomes were analyzed.

**RESULTS:**

The mean gestational age at diagnosis was 21±3.83 weeks. Congenital talipes equinovarus was bilateral in 60.3% of cases. Isolated congenital talipes equinovarus was present in 30.8% of cases, while 69.2% had associated anomalies, most commonly neural tube defects (32.6% of non-isolated cases). Of the 46 cases (59%) that underwent genetic analysis, 13 (28.3%) revealed abnormalities, representing 16.7% of the total cohort. Termination of pregnancy occurred in 59% of cases due to associated anomalies or genetic abnormalities. Thirty pregnancies resulted in live birth, with a prenatal diagnosis accuracy of 90%. The three false-positive cases were associated with oligohydramnios or early gestation. Postnatally, most live-born cases with confirmed congenital talipes equinovarus (77.8%) required serial casting and surgery.

**CONCLUSION:**

The prognosis for prenatally diagnosed congenital talipes equinovarus is excellent in isolated cases, supported by the absence of genetic abnormalities in this group. However, the high frequency of associated anomalies drastically alters prognosis, frequently leading to pregnancy termination and highlighting the critical need for comprehensive prenatal screening and multidisciplinary counseling.

## INTRODUCTION

Congenital talipes equinovarus (CTEV), or clubfoot, ranks among the most prevalent congenital musculoskeletal disorders. The deformity presents with a classic triad of foot positioning: equinus (plantar flexion), varus (inversion of the heel), and medial adduction and supination of the forefoot^1^. The reported global prevalence of this condition is approximately 1–2 cases per 1,000 live births^1^.

The precise pathogenic mechanisms underlying CTEV remain incompletely understood. Current evidence suggests a multifactorial origin, where genetic susceptibility interacts with environmental influences during the second trimester, as opposed to a primary embryonic malformation^2^. Various pathophysiological pathways have been hypothesized, implicating potential neurological, vascular, muscular, connective tissue, and skeletal abnormalities^2^. Established risk factors for CTEV, which demonstrates a 2:1 male-to-female predominance, encompass genetic predisposition, male sex, primiparity, early amniocentesis (conducted around 11–12 weeks of gestation), maternal diabetes mellitus, and maternal smoking^2^. The recurrence risk for siblings of an affected individual is estimated at 2–4%, and this likelihood escalates in proportion to the number of additional affected relatives within the family^2^.

Ultrasonography has been shown to have a high sensitivity of up to 81% in diagnosing CTEV, although the false positive rate is approximately 14%^3^. CTEV is detected unilaterally in 30–40% of cases and bilaterally in 60–70%^4^. While CTEV can present as an isolated deformity in approximately 50–70% of cases, it can also be a finding of chromosomal abnormalities and other genetic syndromes in 30–50% of cases^4^. In some cases associated with intrauterine factors that restrict fetal movement, such as oligohydramnios, multiple pregnancies, and uterine malformations, referred to as “positional talipes equinovarus,” spontaneous resolution of the deformity can be observed^4^.

Nevertheless, detailed data on the spectrum and prognostic impact of associated anomalies, particularly from tertiary referral centers, are essential to refine prenatal counseling and management strategies. This study aims to present a decade of experience from a tertiary center in the Black Sea Region, with a specific focus on the spectrum of associated anomalies, genetic findings, and how the “isolated vs. non-isolated” distinction critically shapes pregnancy outcomes and postnatal management.

## METHODS

A total of 78 cases referred to Samsun Ondokuz Mayıs University Perinatology Clinic with suspected fetal anomaly between 2013 and 2023 and diagnosed prenatally with CTEV were included in this retrospective cohort study. This study received ethical clearance (Protocol No: B.30.2.ODM.0.20.08/605-2025/468) from the Ondokuz Mayıs University Faculty of Medicine Ethics Committee. Throughout the research, patient confidentiality and personal data security were scrupulously maintained.

**Figure 1:**
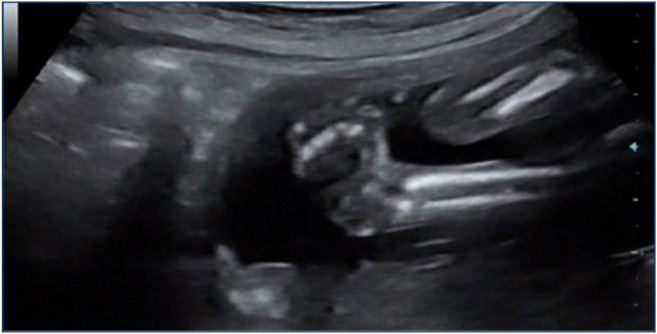
Ultrasonographic appearance of congenital talipes equinovarus: the plantar surface of the fetal foot is observed in the same sagittal plane as the tibia and fibula.

The prenatal diagnosis of CTEV was established by qualified perinatologists following the International Society of Ultrasound in Obstetrics and Gynecology guidelines^5^. The key diagnostic criterion was the simultaneous visualization of the fetal foot’s plantar surface aligned in a single sagittal plane with both the tibia and fibula (Figure 1).

CTEV was classified as unilateral if it affected one foot and bilateral if it affected both feet. Cases without any additional structural or chromosomal anomalies were defined as isolated CTEV, while cases with associated anomalies were defined as non-isolated CTEV. A multidisciplinary team, including specialists in perinatology, pediatrics, and medical genetics, evaluated all cases. In accordance with national regulations, the option of pregnancy termination was discussed with families when severe anomalies were identified.

All families were offered invasive prenatal testing after counseling. Amniocentesis samples were analyzed by high-resolution G-banded karyotyping according to ISCN 2020 standards^6^. When indicated, FISH or whole-exome sequencing (WES) was performed for detailed evaluation.

Demographic characteristics, ultrasonography findings, genetic test results, pregnancy outcomes, and postnatal follow-up data were collected retrospectively from the hospital information management system. The prenatal CTEV diagnosis was confirmed for all live-born newborns by a physical examination performed by a neonatologist within the first 24 h after birth. Postnatal prognosis and treatment information for cases that were delivered or followed up outside our institution were recorded using a standardized questionnaire via telephone interviews. Cases that underwent surgical operation following casting treatment were categorized as having received surgical-casting treatment, while cases treated only with casting and orthosis were included in the Ponseti treatment group.

The data were analyzed in International Business Machines Statistical Package for the Social Sciences Statistics (Version 28.0). In descriptive statistics, categorical variables are expressed as count (n) and percentage (%), and continuous variables as either mean±standard deviation or median (interquartile range), based on their distributional properties. Associations between categorical variables were evaluated using the chi-square test, with Fisher’s exact test employed when appropriate. A p-value below 0.05 was deemed to indicate statistical significance.

## RESULTS

A total of 78 cases prenatally diagnosed with CTEV at Ondokuz Mayıs University Perinatology Clinic between 2013 and 2023 were included in the study. The demographic and clinical characteristics of the cases are summarized in Table 1.

**Table 1:** Demographic and clinical characteristics of the prenatally diagnosed congenital talipes equinovarus cohort (n=78).

Characteristic	Mean±SD, n (%)
Mean maternal age at diagnosis	27±6.16 years
Mean gestational age at diagnosis	21±3.83 weeks
CTEV laterality	
Bilateral	47 (60.3%)
Unilateral	31 (39.7%)
Type of CTEV	
Isolated CTEV	24 (30.8%)
Non-isolated (with additional anomalies)	54 (69.2%)
Gender distribution	
Male	51 (65.4%)
Female	27 (34.6%)
Genetic analysis		
Cases with karyotyping performed	46 (59.0%)
Abnormal karyotype	13 (28.3% of those tested; 16.7% of total cohort)^*^
Normal karyotype	33 (71.7% of those tested; 42.3% of total cohort)
Pregnancy outcomes				
Termination of pregnancy	46 (59.0%)
Fetal demise	1 (1.3%)
Postpartum death	1 (1.3%)
Live births	30 (38.4%)
Mean gestational age at birth	37.8±2.5 weeks
Diagnostic accuracy (live)		
Confirmed postnatal diagnosis	27 (90%)
False positive (no CTEV at birth)	3 (10%)
Postnatal follow up (month)	68 months (27–130 months)
Postnatal management of live births with CTEV (n=27)	
Surgical ıntervention and casting	21 (77.8%)
Ponseti method (serial casting and orthosis)	6 (22.2%)

^*^Percentage of those tested/percentage of entire cohort. CTEV: congenital talipes equinovarus.

The mean maternal age at diagnosis was 27±6.16 years, and the mean gestational week was 21±3.8 weeks. CTEV was bilateral in 47 cases (60.3%) and unilateral in 31 (39.7%). CTEV was isolated in 24 cases (30.8%), while associated anomalies were present in 54 (69.2%). Of the fetuses, 51 (65.4%) were male, and 27 (34.6%) were female.

Genetic analysis was performed on 46 cases (59%), and a genetic abnormality was detected in 13 (16.7% of the entire cohort). The spectrum of these genetic abnormalities was as follows: Trisomy 21 (n=5), Trisomy 18 (n=4), 47,XYY Syndrome (n=1), DiGeorge Syndrome (22q11.2 deletion) (n=1), and partial trisomy 16q22.1-qter/partial monosomy 16p13.3-pter (n=1). In one case referred for WES, a likely pathogenic de novo heterozygous mutation (c.4114+1G>A) was identified in the MED13L gene (n=1).

Among the 54 non-isolated CTEV cases, the spectrum of associated anomalies is detailed in Table 2.

**Table 2:** Associated structural and genetic anomalies in non-isolated congenital talipes equinovarus cases (n:54).

System/category	Associated anomaly	n
Genetic/syndromic conditions	Trisomy 21	5
	Trisomy 18	4
	47,XYY syndrome	1
	22q11.2 deletion (DiGeorge syndrome)	1
	Partial trisomy 16q/partial monosomy 16p	1
	MED13L mutation	1
Central nervous system	Neural tube defect	15
	Severe ventriculomegaly	2
	Dandy-Walker malformation	1
	Rhombencephalosynapsis	1
	Agenesis of the corpus callosum	1
Cardiovascular system	Aortic coarctation	1
	Ventricular septal defect	1
Thoracic	Congenital diaphragmatic hernia	1
Abdominal wall	Omphalocele	1
Renal/genitourinary	Bilateral renal dysplasia	2
	Polycystic kidney disease	1
	Unilateral multicystic dysplastic kidney	1
	Ectopic kidney	1
Musculoskeletal/movement disorders	Fetal akinesia deformation sequence (FADS)	3
	Radial ray malformation	1
Neoplasm	Sacrococcygeal teratoma	1
Craniofacial	Cleft lip and palate	1
Lymphatic system	Cystic hygroma	1
Hydrops/fluid disorders	Non-immune hydrops fetalis	3
Soft markers	Choroid plexus cyst	1
	Renal pyelectasis	2
	Single umbilical artery	2

Note: Values are presented as absolute numbers (n). Some fetuses presented with more than one associated anomaly; therefore, the total number of anomalies exceeds the number of non-isolated CTEV cases.

Forty-six pregnancies (59%) were terminated due to severe associated structural and/or genetic abnormalities. Among these cases, central nervous system anomalies were the most frequently observed associated findings, with neural tube defects detected in 15 cases (27.8% of non-isolated cases), followed by severe ventriculomegaly (n=2, 3.7%), Dandy-Walker malformation (n=1), rhombencephalosynapsis (n=1), and agenesis of the corpus callosum (n=1), as summarized in Table 2.

Cardiovascular anomalies were less frequent and included aortic coarctation in one case. Thoracic and abdominal wall anomalies comprised congenital diaphragmatic hernia (n=1) and omphalocele (n=1), respectively. Renal and genitourinary system anomalies included bilateral renal dysplasia (n=2) and polycystic kidney disease (n=1).

Musculoskeletal and movement disorders included fetal akinesia deformation sequence in three cases. Other associated anomalies consisted of sacrococcygeal teratoma (n=1), cleft lip and palate (n=1), cystic hygroma (n=1), and non-immune hydrops fetalis (n=1).

One of two cases with CTEV and associated hydrops fetalis resulted in intrauterine exitus at 20 weeks of gestation, while the other case resulted in postnatal death on the first day. Thirty pregnancies (38.4%) resulted in live birth at a mean gestational age of 37±2.5 weeks. The prenatal CTEV diagnosis was postnatally confirmed in 27 of the 30 live-born cases (90%), while no CTEV was observed postnatally in three cases (10%), which were assessed as positional talipes equinovarus. These three false-positive cases consisted of one twin pregnancy and two cases followed for oligohydramnios.

Treatment approaches were evaluated for the 27 cases with a confirmed CTEV diagnosis and available postnatal follow-up. Serial casting and surgical treatment were applied to 21 (77.8%) of these cases, while the Ponseti method (serial casting and orthosis) was applied to 6 (22.2%). The median postnatal follow-up time for the cases was 68 months.

The relationship between the laterality of CTEV (unilateral/bilateral) and its isolated status was assessed using the chi-square test, and no statistically significant relationship was found [χ^2^(1)=0.107, p=0.744]. Similarly, no significant relationship was found between the laterality of CTEV and the presence of an abnormal karyotype in the 46 cases that underwent karyotype analysis [χ^2^(1)=0.839, p=0.360].

However, when the frequencies of genetic abnormality in isolated and non-isolated CTEV cases were compared using Fisher’s Exact Test, a statistically significant difference was found between the two groups (p=0.009). All cases with genetic abnormality (n=13) were in the non-isolated CTEV group, while no genetic abnormality was detected in any of the isolated CTEV cases (0/14).

## DISCUSSION

This retrospective cohort study evaluated the demographic characteristics, associated anomalies, genetic results, and clinical outcomes of 78 CTEV cases diagnosed prenatally at a tertiary center in the Black Sea Region. Our findings reiterate the critical importance of prenatal diagnosis, genetic evaluation, and a multidisciplinary approach in the management of CTEV.

The presence of associated anomalies in 69.2% of the cases in our series indicates that CTEV can often be part of a more complex fetal anomaly profile. This rate is higher than the reports of 30–50% in the literature but is similar to the report by Sucu et al. of 70.3%^4,7^. This higher rate is likely attributable to the referral-based nature of our center, which manages a higher proportion of complex fetal anomalies. The high rate of pregnancies resulting in termination (59%) also demonstrates the prognostic implications of these non-isolated cases and their decisive role in families’ termination decisions.

All abnormal genetic results in our cohort were confined exclusively to the non-isolated CTEV group (100%), while no chromosomal anomalies were detected in isolated cases (p=0.009). This finding underscores the strong prognostic relevance of associated anomalies in fetuses with CTEV. In line with the systematic review and meta-analysis by Mascio et al., non-isolated CTEV was associated with a markedly increased risk of chromosomal abnormalities and adverse outcomes, whereas isolated cases were largely linked to favorable prognoses despite a low residual risk reported in the literature^8^. These data highlight the critical importance of comprehensive fetal anomaly assessment after prenatal CTEV diagnosis, supporting reassuring counseling in truly isolated cases and strongly advocating invasive prenatal genetic testing when additional anomalies are present. Trisomy 21 and Trisomy 18 were the most frequently observed chromosomal abnormalities in our study, further confirming their established association with CTEV^8,9^.

In one case in our cohort, a MED13L gene heterozygous mutation was identified in association with mild ventriculomegaly and bilateral CTEV, which is related to the rare autosomal dominant “Impaired intellectual development and distinctive facial features with or without cardiac defects (Mental Retardation, Facial Dysmorphism, and Cardiac Defects [MRFACD]; Asadollahi-Rauch Syndrome).” This syndrome causes intellectual disability, speech delay, dysmorphic facial features, and skeletal and cardiac anomalies^9^. Similar to that study, our case also had ventriculomegaly in addition to CTEV. This finding is significant as it demonstrates the diagnostic yield of advanced genetic tests, such as WES, beyond genetic analysis in non-isolated CTEV cases.

In another case in our cohort, the association of aortic coarctation and bilateral CTEV was accompanied by the rare cytogenetic finding of partial trisomy (16q22.1-qter)/partial monosomy (16p13.3-pter). The source of this unbalanced rearrangement was a balanced inversion in the father’s chromosome 16 [46,XY, inv(16)(p13.3q22)]. Trisomy 16q is generally associated with a multisystemic phenotype, including intellectual disability, intrauterine growth restriction, brain and cardiac defects, and some skeletal anomalies^10^. Deletion of the short arm (16p) is associated with developmental delay and dysmorphic features. The cardiac anomaly and CTEV in our case were considered likely due to the 16q trisomy. It is probable that CTEV manifested in our case as an expression of this complex genotype. This situation highlights that multifactorial anomalies like CTEV can also arise as a consequence of unexpected chromosomal irregularities and underscores the important role of standard karyotyping and array-comparative genomic hybridization in detecting unbalanced products of balanced translocations.

The Ponseti method has revolutionized CTEV treatment and has been adopted worldwide over the last few decades^11^. However, most reported outcomes of the Ponseti method are short or medium-term^12^. In our study, the treatment processes of our 27 live-born and followed cases were evaluated in the postnatal period. The preference for surgery and casting over the Ponseti method in these cases may be due to the long follow-up period of our cases, their management not being centralized in a single facility, or the cases predominantly comprising complex/severer forms. A significant proportion of cases were observed to be directed to private hospitals to ensure meticulous follow-up and treatment, due to reasons such as high patient volume in state hospitals, difficulties in scheduling appointments, and challenges in maintaining continuity in physiotherapy and casting procedures. This finding underscores a critical gap in care coordination and highlights the urgent need for established, centralized clinics to ensure consistent application of the Ponseti method and optimize long-term outcomes.

Several limitations should be considered when interpreting the results of this study. First, the retrospective design poses a potential risk of selection bias in data collection and may have resulted in missing data for some cases. A second important limitation is that the data were obtained from a single tertiary referral center; this may lead to a case population with a tendency toward more complex presentations, potentially limiting the generalizability of the results to the general population.

Third, the lack of a standardized, centralized follow-up protocol for postnatal orthopedic care led to fragmented data collection on treatment details and long-term functional outcomes. This highlights an area for improvement in the management pathway for prenatally diagnosed congenital anomalies.

In conclusion, a prenatal diagnosis of CTEV can often be a clue to an underlying serious constellation of anomalies or a chromosomal disorder. Our study demonstrated that, in addition to common aneuploidies such as Trisomy 21 and 18, rare syndromic conditions like MED13L mutation and chromosome 16 irregularities can also be associated with CTEV. Therefore, detailed fetal ultrasonography and genetic counseling are essential in all cases where prenatal CTEV is detected.

In contemporary practice, beyond standard karyotyping, the use of high-resolution genetic tests such as chromosomal microarray and WES is increasingly vital for providing prognostic information and accurate genetic counseling.

In the postnatal period, ensuring access to standardized treatment protocols like the Ponseti method within a centralized, multidisciplinary follow-up program is crucial to optimize functional outcomes for these children.

## Data Availability

The datasets generated and/or analyzed during the current study are available from the corresponding author upon reasonable request.
